# Associations between friendship characteristics and HIV and HSV‐2 status amongst young South African women in HPTN‐068

**DOI:** 10.1002/jia2.25029

**Published:** 2017-12-29

**Authors:** Elizabeth Fearon, Richard D Wiggins, Audrey E Pettifor, Catherine MacPhail, Kathleen Kahn, Amanda Selin, F Xavier Gómez‐Olivé, Sinéad Delany‐Moretlwe, Estelle Piwowar‐Manning, Oliver Laeyendecker, James R Hargreaves

**Affiliations:** ^1^ Department of Social and Environmental Health Research London School of Hygiene and Tropical Medicine London UK; ^2^ Department of Social Science UCL Institute of Education University College London London UK; ^3^ Department of Epidemiology Gillings School of Global Public Health University of North Carolina Chapel Hill NC USA; ^4^ Wits RHI University of the Witwatersrand Johannesburg South Africa; ^5^ School of Health and Society University of Wollongong Wollongong NSW Australia; ^6^ Medical Research Council/Wits University Rural Public Health and Health Transitions Research Unit (Agincourt) School of Public Health Faculty of Health Sciences University of the Witwatersrand Johannesburg South Africa; ^7^ Centre for Global Health Research Umeå University Umeå Sweden; ^8^ INDEPTH Network Accra Ghana; ^9^ Department of Pathology Johns Hopkins University School of Medicine Baltimore MD USA; ^10^ Division of Infectious Diseases Johns Hopkins University School of Medicine Baltimore MD USA; ^11^ Division of Intramural Research National Institute of Allergy and Infectious Diseases National Institutes of Health Bethesda MD USA

**Keywords:** HIV, young women, adolescents, social networks, friendships, peer influence, social norms, HSV‐2, South Africa

## Abstract

**Introduction:**

Prevalence of HIV among young women in South Africa remains extremely high. Adolescent peer groups have been found to be an important influence on a range of health behaviours. The characteristics of young women's friendships might influence their sexual health and HIV risk via connections to sexual partners, norms around sexual initiation and condom use, or provision of social support. We investigated associations between young women's friendships and their Herpes Simplex Virus Type 2 (HSV‐2) and HIV infection status in rural South Africa.

**Methods:**

Our study is a cross‐sectional, egocentric network analysis. In 2011 to 2012, we tested 13‐ to 20‐year‐old young women for HIV and HSV‐2, and collected descriptions of five friendships for each. We generated summary measures describing friend socio‐demographic characteristics and the number of friends perceived to have had sex. We used logistic regression to analyse associations between friend characteristics and participant HIV and HSV‐2 infection, excluding likely perinatal HIV infections.

**Results:**

There were 2326 participants included in the study sample, among whom HIV and HSV‐2 prevalence were 3.3% and 4.6% respectively. Adjusted for participant and friend socio‐demographic characteristics, each additional friend at least one year older than the participant was associated with raised odds of HIV (odds ratio (OR) = 1.37, 95% CI 1.03 to 1.82) and HSV‐2 (adjusted OR=1.41, 95% CI 1.18 to 1.69). Each additional friend perceived to have ever had sex also raised the odds of HIV (OR = 1.29, 95% CI 1.03 to 1.63) and HSV‐2 (OR=1.18, 95% CI 1.03 to 1.35).

**Discussion:**

We found good evidence that a greater number of older friends and friends perceived to have had sex were associated with increased risk for HSV‐2 and HIV infection among young women.

**Conclusions:**

The characteristics of young women's friendships could contribute to their risk of HIV infection. The extent to which policies or programmes influence age‐mixing and young women's normative environments should be considered.

## 1 Introduction

South Africa has a severe HIV epidemic with young women at high risk for infection 1, 2. While young women's sexual behaviours can influence their HIV risk 3, the limited impact of individual behavioural interventions on HIV incidence has pointed to the importance of structural factors that characterise and shape young women's social environments 4, 5, 6, 7, 8. Adolescents’ peers, who make up much of their day‐to‐day social environment, could be important links in causal chains from structural drivers of HIV risks to proximate HIV exposures.

Peer relationships grow in importance as children move into adolescence 9, 10, 11. They may serve as conduits for dominant norms about gender and sexuality, as well as for information and resources and as the social contacts through which young women form sexual partnerships. Research from other populations has found that characteristics of adolescents’ peers and friends can affect a range of behaviours, including sexual behaviour 12, 13, 14, 15. There are different mechanisms by which such influence could occur 16. Young women who believe that more of their friends are sexually active might become sexually active themselves. Previous research has found such perceived behaviour, often referred to as “descriptive norms,” can encourage people to align their behaviour accordingly, even when such perceptions are inaccurate 17, 18. The socio‐demographic characteristics of friends could affect the kinds of acquaintances and sexual partners a young woman is likely to meet, as well as the sexual behaviours she perceives to be normative. A previous study from the Western Cape in South Africa has found that estimated exposure to older classmates over time, common when young people repeat grades at school, was associated with sexual debut and having older sexual partners in young women 19. Friends serve as social connections, introducing young people to partners and helping to facilitate relationships 20. Because social relationships tend to exhibit a high level of homophily (similarity), male friends, friends out of school and older friends might be more likely to connect young women to older sexual partners than younger female friends 21.

This study explores the possible role of adolescent girls’ friends in their risk of acquiring HIV. While qualitative research from South Africa suggests that peers might be influential 6, 20, 22, 23, the quantitative evidence base is weak and there is no study that uses HIV as an outcome 24.The baseline survey 25 from HPTN 068: Effect of Cash Transfer for the Prevention of HIV among Young South African Women provided an opportunity to examine the evidence for these hypotheses about the associations between friendship characteristics and HIV and HSV‐2 status amongst 13‐ to 20‐year‐old young women in rural South Africa.

## 2 Methods

### 2.1 Population and setting

Participants were 13‐ to 20‐year‐old unmarried young women enrolled in grades 8 to 11 in school and recruited between March 2011 and December 2012 for HPTN 068. The trial investigated the effect of conditional cash transfers (CCT) on young women's school attendance and on HIV acquisition 26.

Participants resided in the Agincourt health and socio‐demographic surveillance system (HDSS) site 27 including 28 villages in a deprived and densely populated, but rural area in Mpumalanga province in north‐east South Africa. HIV prevalence among individuals over 15 in the site is 19.4% of women and 10.6% of men. Prevalence by age rises steeply from 5.5% of young women aged 15 to 19 to 27% among women aged 20 to 24 years 28.

### 2.2 Data collection

Fieldworkers visited households identified in the annual demographic census as likely to house eligible young women in order to confirm eligibility, explain the study and obtain informed consent (assent for under 18s) from one eligible young woman per household and a parent/guardian. The parent/guardian then completed a survey with information about the household and the young woman was given a time to attend a “weekend camp” at a nearby community venue or study office 26. Here, she completed her baseline survey, HIV/HSV‐2 testing and pre and post‐test counselling and randomisation to study arm.

The young woman's survey, conducted in either English or Shangaan, included sections on socio‐demographic characteristics, education, sexual behaviours, characteristics of up to three previous sexual partners and a module on friendships. Participants completed the survey alone using Audio Computer Assisted Self Interview (ACASI) for most modules to reduce social desirability bias 29. The friendship module was delivered by trained interviewers using Computer Assisted Personal Interview (CAPI) because the module structure was relatively complex and less sensitive in nature.

### 2.3 Measures

#### 2.3.1 Friendship exposures

Young women were asked to think of five of their closest friends – their “friendship nets.” Personal identifiers were not collected. They described these friends and their relationship to them in terms of their socio‐demographic attributes, where and how often they saw them, how long they had known them, their perceptions of their sexual behaviour, and communication with them about sex and HIV. The questionnaire was translated and back‐translated and interpretation of “friend” in Shangaan was checked. We did not attempt to impose a definition of “friend” as we wanted to capture the diversity in friendship types that might exist.

For this analysis, we have used a “personal network exposure” approach to capture the influence of the network (also referred to as an ego‐net) 30, 31, 32, 33, rather than examining the effects of each friend separately because it is a better measure of the overall friendship environment a young woman is exposed to. Thus, friendship exposures for each participant were treated either as values between 0 and 5, or as binary variables (“has at least one friend”) for rarer friendship characteristics. Young women were excluded if they refused to give a response to one of the friendship characteristics for more than three friends.

The main friendship variables of interest to this study included: the number of friends perceived to have ever had sex; the number of friends who were more than one year older than the participant; whether the participant reported at least one friend not attending school; and whether the participant reported at least one male friend. Because approximately one‐quarter of friends were also reported to be blood relatives, we included number of friends who were also blood relatives in the analysis.

#### 2.3.2 Outcomes

Two different, site‐validated, HIV rapid tests, the Determine™ HIV‐1/2 (Alere Medical Co., Ltd, Matsudo‐shi, Chiba, Japan) and FDA‐cleared Uni‐gold Recombigen HIV test (Trinity Biotech plc, Bray, Co. Wicklow, Ireland), were conducted in parallel. If one or both of the tests was reactive, a CD4 count and a western blot confirmatory test were conducted. Participants were considered to be HIV seropositive if western blot test was positive. HSV‐2 testing was completed using the Kalon assay (Herpes Simplex Virus Type 2 IgG ELISA; Kalon Biologics Ltd, Guildford, UK) with an index cutoff of 1.5.

### 2.4 Analysis

We examined differences between the sample population and those excluded for missing data. We summarised socio‐demographic characteristics of the participants alongside the characteristics of their friendship networks and their HIV and HSV‐2 status. To test whether outcomes varied by village or school, we fit two‐level logistic regression models containing only the outcome (null effects models) with random effects by school or village to examine whether the proportion of the variance explained by village or school was greater than would be expected by chance by inspecting the estimated intraclass correlation coefficients under a modified χ^2^ 34.

The characteristics of participants, including those of their households, could plausibly affect both what kind of friends they have and their risk of HIV and HSV‐2 35. We adjusted for the participants’ age in years, school grade, whether participants’ parents were alive, relative household socio‐economic position (SEP), and parent's education. It was possible that friend characteristics could confound each other, such as confounding between friend age and perceived sexual activity. However, to avoid over‐adjusting, we first entered the socio‐demographic characteristics of friends (Model 1) and then added the number of friends perceived to have had sex to the model (Model 2). We assessed the evidence for associations using likelihood ratio tests to compare models.

To assess whether participant and friendship net characteristics might operate differently in younger (13 to 15 years old) versus older participants (16 to 20 years old), we used likelihood ratio tests to compare models with and without interaction terms.

It is plausible that some HIV‐positive young women in the study were perinatally infected. While there has been an absence of long‐term cohort studies to observe survival among adolescents born and infected prior to availability of antiretroviral treatment (ART), recent studies suggest that 20% to 30% could survive to at least age 10 36, 37, 38, 39, 40, 41. This could represent a substantial proportion of prevalent HIV cases, particularly among younger adolescents. On this basis, given the rapidly rising HIV incidence amongst antenatal attendees 42, 43 in South Africa during the period in which these young women were born (1991 to 1999), and given our findings presented in the Additional File, we chose in our primary analyses for HIV outcome to exclude young women who were HIV positive, yet reported never having had sex, n=36. (They were not excluded from HSV‐2 analyses). Evidence for this decision is presented in an Additional File, but associations with HIV discussed in this paper otherwise refer to young women excluding those judged to have been perinatally infected.

### 2.5 Ethics

The HPTN 068 study trial has ethical approval from the Ethics Committees of the University of North Carolina, the University of the Witwatersrand, Mpumalanga Province Health Research and Ethics Committee and the London School of Hygiene and Tropical Medicine (LSHTM). The analysis presented here has additional approval from the LSHTM.

## 3 Results

### 3.1 Participant characteristics and those of their friends

**Figure 1:**
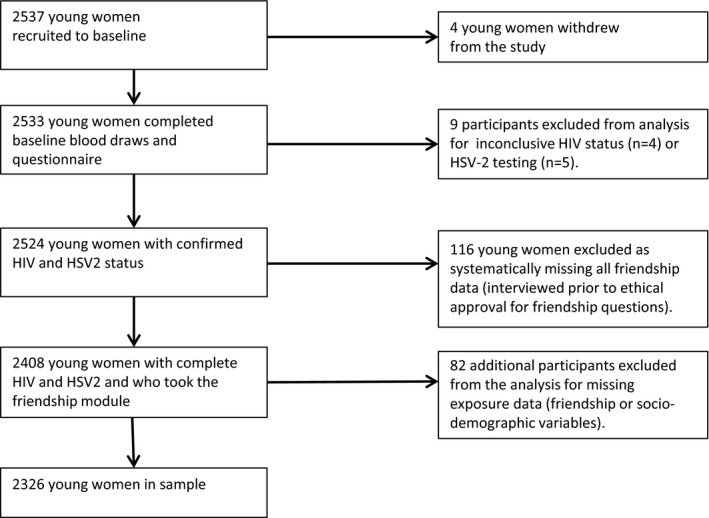
Flow diagram of study sample.

There were 2537 young women recruited to the HPTN 068 study, of whom 2326 were retained for this study, (Figure 1). Four young women were withdrawn from the study, four were excluded because HIV infection status could not be determined due to incomplete or inconclusive test results, and five were excluded because their HSV‐2 tests were inconclusive. There were 116 participants from one village who did not complete the friendship module as this was delayed in ethical review and not yet included in the survey. We did not find differences in their socio‐demographic characteristics, HIV or HSV‐2 prevalence from other participants (not shown here).

Of the 2326 young women included in the study, mean age was 15.5 years old (Table 1). For 32.0% of young women, either their father, mother or both parents had died or their vital status was unknown. Parents were reported to have a mix of educational attainments, with 16.4% of mothers and 17.0% of fathers reported to have had no schooling.

**Table 1:** Characteristics of participants and their friendship nets, n=2326

Participant characteristics	n	*%*
Participant age in years	15.5 (mean)	*15 (median)*
School grade		
8	598	25.7
9	628	27.0
10	631	27.1
11	469	20.2
Orphanhood status		
Both alive	1581	68.0
Father died/unknown	465	20.0
Mother died/unknown	143	6.1
Both died/unknown	137	5.9
Father's highest level of education		
No school	396	17.0
Attended primary (complete and incomplete)	421	18.1
Attended secondary but did not complete	431	18.5
Completed secondary school plus	649	27.9
Don't know	429	18.4
Mother's highest level of education		
No school	381	16.4
Attended primary (complete and incomplete)	511	22.0
Attended secondary but did not complete	636	27.3
Completed secondary school plus	597	25.7
Don't know	201	8.6
Number of friends perceived to have had sex		
0	913	39.3
1	330	14.2
2	254	10.9
3	261	11.2
4	260	11.2
5	308	13.2
Number of friends >1 year older		
0	1085	46.6
1	621	26.7
2	337	14.5
3	192	8.3
4	64	2.8
5	27	1.2
At least one male friend		
Yes	298	12.8
At least one friend out of school		
Yes	451	19.4
Number of friends who are relatives		
0	933	40.1
1	680	29.2
2	433	18.6
3	178	7.7
4	72	3.1
5	30	1.3
HIV status		
Positive	76	3.3
HSV‐2 status		
Positive	106	4.6

Of the 2326 young women in the sample, there were 76 who tested positive for HIV (3.3%, 95% CI 2.5 to 4.0), 36 of whom reported never having had sex and were therefore excluded from further analysis investigating HIV. The HSV‐2 prevalence was 4.6%, 95% CI 3.7 to 5.4 (106 participants). There was no evidence that prevalence of HIV and HSV‐2 varied significantly across schools or villages, ICCs<0.01.

Over half of participants reported that at least one of their five reported friends was more than one year older (53.4%). Only 12.8% of participants reported a male friend, and 80.6% reported that all their friends attended school. There were 59.9% who included at least one friend who was also a blood relative. For 39.3% of young women, no friends were perceived to have had sex, while 13.2% thought that all of their friends had sex.

### 3.2 Associations with HIV and HSV‐2, adjusted for participant age

HIV‐infected young women were on average older than HIV uninfected young women (mean age 17.4 vs. 15.5 years), and the odds of being HIV infected increased with every additional year of age (unadjusted OR 1.89; 95% CI 1.57 to 2.28) (Table 2). There was no association between HIV status and grade at school, whether parents were alive, household SEP, and mother's or father's education when adjusted for age.

**Table 2:** Associations adjusted only for participant age with HIV (excluding those reporting never having had sex, n=2290) and HSV‐2 (n=2326)

	HIV, n=2290						HSV‐2, n=2326					
	HIV positive n, %		OR	95% CI		*p* value	HSV‐2+ n, %		OR	95% CI		*p* value
Each additional year in age	17.4 mean HIV positive	15.5 mean HIV negative	1.89	1.57	2.28	<0.001	15.4 mean HSV‐2‐	17.0 mean HSV‐2+	1.73	1.54	1.95	<0.001
School grade												
8	2/577	0.3	1.00			0.618	9/598	1.5	1.00			0.597
9	8/622	1.3	2.11	0.44	10.18		15/628	2.4	1.02	0.43	2.37	
10	9/626	1.8	1.32	0.27	6.45		39/631	6.2	1.48	0.67	3.28	
11	18/465	4.1	1.84	0.38	9.07		43/469	9.2	1.43	0.62	3.33	
Orphanhood												
Parents alive	22/1565	1.4	1.00			0.277	66/1581	4.2	1.00			0.564
Mother only alive	9/457	2.0	1.25	0.56	2.76		22/465	4.7	1.03	0.62	1.71	
Father only alive	3/140	2.1	1.43	0.41	4.97		10/143	7.0	1.71	0.83	3.50	
Neither parent alive	6/128	4.7	2.78	1.08	7.20		8/137	5.8	1.19	0.55	2.59	
Household SEP												
First quintile	10/452	2.2	1.00			0.631	33/461	7.2	1.00			0.010
Second	7/454	1.5	0.74	0.27	2.00		12/460	2.6	0.36	0.18	0.71	
Third	6/454	1.3	0.67	0.24	1.89		16/461	3.5	0.51	0.27	0.95	
Fourth	11/461	2.4	1.36	0.56	3.30		20/468	4.3	0.68	0.38	1.23	
Fifth	6/469	1.3	0.79	0.28	2.23		25/476	5.3	0.95	0.54	1.66	
Mother's education												
No school	8/378	2.1	1.00			0.458	24/381	6.3	1.00			0.669
Attended primary but not completed	13/502	2.6	1.38	0.56	3.42		25/511	4.9	0.86	0.47	1.55	
Completed primary, some high school	10/629	1.6	0.95	0.37	2.46		30/636	4.7	0.91	0.52	1.61	
Completed high school	4/582	0.7	0.52	0.15	1.78		21/597	3.5	0.84	0.45	1.56	
Do not know	5/199	2.5	1.38	0.44	4.38		6/201	3.0	0.51	0.20	1.29	
Father's education												
No school	8/390	2.1	1.00			0.728	26/396	6.1	1.00			0.055
Attended primary but not completed	6/415	1.4	0.75	0.25	2.21		19/421	5.0	0.73	0.39	1.37	
Completed primary, some high school	5/428	1.2	0.86	0.27	2.70		19/431	4.4	0.91	0.49	1.70	
Completed high school	11/635	1.7	1.38	0.54	3.55		14/649	2.3	0.45	0.23	0.88	
Do not know	10/422	2.4	1.29	0.50	3.37		28/429	6.8	1.12	0.63	1.98	
Each additional friend perceived to have had sex	1.8 mean HIV negative	3.6 mean HIV positive	1.36	1.09	1.68	0.004	1.7 mean HSV‐2 ‐	3.2 mean HSV‐2 +	1.25	1.10	1.41	<0.001
Each additional friend ≥1 year older	1.0 mean HIV negative	1.4 mean HIV positive	1.44	1.14	1.83	0.005	1.0 mean HSV‐2 ‐	1.3 mean HSV‐2 +	1.40	1.20	1.63	<0.001
No male friends	31/2000	1.6	1.00			0.135	88/2028	*4.3*	1.00			0.336
≥1 male friend	9/290	3.1	1.86	0.86	4.01		18/298	*6.0*	1.31	0.77	2.24	
All friends in school	23/1851	1.2	1.00			0.112	69/1875	*3.7*	1.00			0.155
≥1 friend out of school	17/439	3.9	1.74	0.89	3.41		37/451	*8.3*	1.38	0.89	2.15	
Each additional friend who is a relative	2.2 mean HIV negative	3.0 mean HIV positive	1.09	0.97	1.23	0.157	2.2 mean HSV‐2 ‐	2.2 mean HSV‐2 +	0.98	0.90	1.06	0.577

As with HIV, HSV‐2 seroprevalence increased with age (OR=1.73, 95% CI 1.54 to 1.95). In contrast to HIV, being HSV‐2 positive was less common among participants from the second and third SEP quintiles compared to the first (OR=0.36, 95% CI 0.18 to 0.71 and OR=0.51, 95% CI 0.27 to 0.95, respectively) and among young women whose fathers had completed high school compared to those whose fathers had no schooling (OR=0.45, 95% CI 0.23 to 0.88).

### 3.3 Friendship socio‐demographic characteristics and associations with HIV and HSV‐2

Young women who were HIV positive reported that a higher number of friends were at least one year older than themselves (mean 1.4 vs. 1.0 of the five friends). Each additional friend at least one year older was associated with increased odds of HIV infection, after adjusting for age, (adjusted OR=1.44, 95% CI 1.14 to 1.83), (Table 2). Controlling for other participant and friend socio‐demographic characteristics, weakened this association only slightly (OR=1.37 95% CI 1.03 to 1.82) (Table 3 Model 1).

**Table 3:** Associations between friendship network characteristics and HIV (excluding those reporting never having had sex, n=2290) and HSV‐2 (n=2326)

	Model 1				Model 2				Model 1				Model 2			
	OR	95% CI		*p* value	OR	95% CI		*p* value	OR	95% CI		*p* value	OR	95% CI		*p* value
Each additional friend perceived to have had sex					1.29	1.03	1.63	0.025					1.18	1.03	1.35	0.014
Each additional friend ≥1 year older	1.37	1.03	1.82	0.036	1.26	0.94	1.69	0.123	1.41	1.18	1.69	<0.001	1.34	1.11	1.61	0.002
No male friends	1.00			0.202	1.00			0.163	1.00			0.295	1.00			0.292
≥1 male friend	1.75	0.77	3.98		1.85	0.81	4.22		1.37	0.77	2.43		1.37	0.77	2.43	
All friends in school	1.00			0.755	1.00			0.851	1.00			0.734	1.00			0.808
≥1 friend out of school	1.13	0.52	2.49		1.08	0.49	2.36		1.09	0.67	1.78		1.06	0.65	1.74	
Each additional friend who is a relative	1.04	0.91	1.19	0.521	1.06	0.92	1.21	0.439	0.94	0.86	1.04	0.216	0.95	0.86	1.04	0.239

All models also adjusted for participant age in years, school grade, whether each parent was alive, household SEP, and mother's and father's education.

Similar results were found for HSV‐2, with the odds of infection increasing for each additional older friend, even after adjustment for age (OR 1.40, 95% CI 1.20 to 1.63) (Table 2) and participant and friend characteristics (OR 1.41 95% CI 1.18 to 1.69, Table 3, Model 1).

There was little evidence for raised odds of HIV among young women with at least one friend out of school (OR=1.74, 95% CI 0.89 to 3.41) and one friend who was male (OR=1.86, 95% CI 0.86 to 4.01) when adjusted for the participant's age (Table 2). Once adjusted for participant and friend socio‐demographic characteristics, the association reduced further (OR=1.13, 95% CI 0.52 to 2.49) in the case of a friend out of school and for an effect of having at least one male friend (OR = 1.75, 95% CI 0.77 to 3.98, Table 3 Model 1). These characteristics were not associated with HSV‐2 status. Having more friends who were relatives was also not found to be associated with either HIV or HSV‐2 (Tables 2 and 3).

### 3.4 Perceived ever sex among friends and associations with HIV and HSV‐2

There was good evidence that perceiving a greater number of friends to have ever had sex was associated with both HIV and HSV‐2 infection. Adjusted for participant age, each additional friend thought to have had sex was associated with raised odds of HIV, (OR=1.36 95% CI 1.09 to 1.68) and for HSV‐2 (OR=1.25 95% CI 1.10 to 1.41). The associations diminished but remained present even after further adjustment for participant and friend socio‐demographic characteristics, including the number of older friends a young woman had. After adjustment, each additional friend perceived to have had sex was associated with 1.29 times the odds of HIV (95% CI 1.03 to 1.63) and 1.18 times the odds of HSV‐2 (95% CI 1.03 to 1.35, Table 3 Model 2).

We found no evidence that associations between friendship characteristics and HIV and HSV‐2 status differed between younger (13 to 15 years) and older (16 to 20 years) participants.

## 4 Discussion

In this large sample from rural South Africa, the majority of young women's friends were also female and in school. Young women who had more friends who were at least one year older than themselves, and those who perceived that more of their friends had sex were more likely to be HSV‐2 positive and to be HIV positive. There was strong evidence for these associations, even when we adjusted for the participant's and friends’ socio‐demographic characteristics.

Our findings are consistent with those from Lam *et al*.'s study in the Western Cape, South Africa, which found that a higher estimated exposure to older school classmates was associated with earlier sexual debut 19. Grade repetition has historically been common in South African schools, particularly among those primarily serving Black South Africans and disadvantaged communities 44, under‐resourced schools, and in Limpopo and Mpumalanga provinces, where our study was set 45. Older friends could lead both to a normative environment encouraging earlier sex and could also serve as social connections to older sexual partners. Taken together, our study and that of Lam *et al*. suggest that high levels of age mixing in settings like schools in which young women form their friendships could have negative effects on the sexual health of young people. However, since 2012 to 2014 changes in the Department of Basic Education's grade progression policy has meant that pupils should only repeat a grade once per “phase” (every three years), so the exposure of young people to older pupils in the same grades and age‐mixing within friendships might now be decreasing 46.

While having a greater number of older friends increased the odds of HSV‐2 and HIV, there was evidence for an independent effect of perceiving more friends to have had sex on HSV‐2. For HIV, much of the association with age could be due to this norm as the strength and evidence for this association diminished when perceived friend sexual activity was added to the model (Table 3, Model 2). There have been other interventions to reduce young people's alcohol consumption via changing norms about the perceived prevalence of drinking behaviour amongst their peers 17, 47, 48. A similar approach could be taken within HIV prevention interventions in this population. Delaying sexual debut in adolescents is a valuable public health initiative with potential for impact beyond the individual.

### 4.1 Strengths and Limitations

Our study had a large sample size and was nested within a randomised control trial and demographic surveillance site with experienced staff and strong oversight of study procedures. Our study did not rely on self‐reported sexual behaviour as outcomes, which are subject to recall and social desirability biases 49, 50. We examined effects of friend characteristics on HIV status directly and found similar associations as with HSV‐2 status, which further strengthens our findings when excluding young women who might have been perinatally infected.

Unlike other studies of peer influence on adolescent sexual behaviour from sub‐Saharan Africa found in a recent systematic review 24, our study collected data about specific friends rather than asking about peers in general. This approach allowed us to better estimate the effects of different friend attributes within each young woman's friendship network, for example controlling for the effect of having older friends when examining the effect of having more friends who were perceived to have had sex. Distinguishing between the effects of different friend characteristics could help to focus on appropriate points for intervention, whether on perceptions of peer behaviours, the social environment in which friendships are formed, or a combination. As neither HIV nor HSV‐2 varied significantly at the school/village level, contextual confounding at this level is unlikely 51.

A limiting factor, however, is that our study was cross‐sectional, and we therefore could not attribute causal associations between participant and friend characteristics. It is possible that young women chose friends who were like themselves and this accounts for the associations seen between friendship net characteristics and HIV and HSV‐2 status 52, 53, 54, 55. It is also possible that young women might not have reported socio‐demographic characteristics of their friends accurately and that social desirability bias could have affected associations between perceived ever sex of friends and HSV‐2 and HIV status.

### 4.2 Further research

Further research into young women's friendships in South Africa, including their formation and dissolution, stability, levels of intimacy, factors associated with status and reputation and their influence vis‐à‐vis parents/caregivers would further benefit the interpretation of our findings.

Some qualitative evidence suggests that sexually active young women risk disapproval from the community and even from young men as not conforming to standards of “good behaviour” 20. On the other hand, research among young women has found that material goods such as toiletries, cosmetics and fashionable clothing that can serve as a motivation for transactional sex were seen as important to avoid social exclusion by one's peers 56, 57. Collecting data to inform the whole connected network of friendships would help to disentangle these seemingly contradictory pressures by exploring associations between HIV risk behaviours and status/popularity, as indicated by centrality within the whole friendship network. Furthermore, an egocentric design allows examination of friend influence only over a “path length” of one, whereas a whole network would allow analysis of clustering of behaviours amongst friends, the extent to which individual young women might “bridge” across diverse normative clusters, and how this structural network position might mediate the influence of norms. Previous research has found some evidence that individuals could be influential on the behaviours of others up to three social connections away, which is not possible to examine egocentrically 58. While there are ethical challenges to identifying friends in order to put together such a connected network 59, 60, link‐tracing network sampling methodologies that would not require participants to name alters in an interview could be promising 61. Distinguishing between peer influence and other possible explanations for the associations seen between friendship characteristics and HIV, as well as between perinatal and sexually transmitted HIV infections, would be facilitated by a longitudinal study design.

HIV prevention research among young women would benefit from assessing how interventions might influence friendship and social networks. Friendships could be investigated as possible mediators, or modifiers, of intervention effects 35. An understanding of peer influence could also be used to improve the targeting of peer‐driven and delivered HIV prevention programmes, as has been shown to be efficacious in anti‐smoking 62, 63, antisubstance use 64 and sexual health promotion 65. Behaviour change interventions might benefit from targeting whole peer groups rather than individuals in isolation of their peers. These peer or friendship groups could be discerned and targeted via a peer‐referral strategy 66, 67.

## 5 Conclusions

Policies and programmes that lead to age mixing within young women's social environments and friendships, including within schools, could increase risk of HSV‐2 and HIV. It may be beneficial for programmes to target norms of perceived sexual activity. We have suggested further research that could additionally strengthen and interrogate our findings. We do not suggest that young women should pick and choose friends on the basis of their characteristics. Rather, our research should be interpreted to suggest that HIV prevention interventions and policies relating to young women should consider their impact on the composition of young women's social environments in which these friendships are formed.

## Competing interests

No author has competing interests to declare.

## Author contributions

EF and JRH conceived the project. EF conducted analyses, led interpretation, and manuscript writing, and was supervised by JRH and RDW. AEP, CM and KK are PI's of the HPTN 068 study and contributed to the study conception, interpretation and drafting of the manuscript. AS project managed the HPTN 068 study and contributed to the manuscript. FXGO was a site leader for the HPTN 068 study and contributed to the manuscript. SDM contributed to the interpretation of findings and to the final drafting of the manuscript. OL and EPM led laboratory analyses and contributed to the manuscript. All authors have read and approved the final manuscript.

## Funding

EF was funded with a Bloomsbury Colleges PhD studentship with fieldwork funding from the London International Development Centre. This work was supported by Award Numbers UM1 AI068619 (HPTN Leadership and Operations Center), UM1AI068617 (HPTN Statistical and Data Management Center) and UM1AI068613 (HPTN Laboratory Center) from the National Institute of Allergy and Infectious Diseases, the National Institute of Mental Health and the National Institute on Drug Abuse of the National Institutes of Health. This work was also supported by NIMH R01 (R01MH087118) and the Carolina Population Center and its NIH Center grant (P2C HD050924). The content is solely the responsibility of the authors and does not necessarily represent the official views of the National Institutes of Health.

## Additional files 1

### Additional File 1. Possible perinatal HIV infections

Additional analyses to investigate the possibility that some HIV infections were due to perinatal infection rather than sexual transmission, which supports their exclusion from the main analyses.

### Background

It was possible that some young women aged 13 to 20 might have HIV not acquired through sexual transmission but through perinatal or breastfeeding transmission from their mothers (vertical transmission). Young women aged 13 to 20 in 2011/2012 would have been born between 1991 and 1999, years of rising incidence of HIV in South Africa and before availability of antiretrovirals for prevention of vertical transmission from mothers to their children in this population 42. As discussed in the main part of this paper, there is recent evidence that a larger proportion of children infected with HIV might have survived into adolescence than previously thought, even without access to ART earlier in their childhoods.

Our hypotheses concerning the role of friendships in HIV risk all posited that friend characteristics were associated with HIV via sexual behaviour or partnership characteristics. If some proportion of young women who were HIV positive did not acquire the infection sexually the estimates for the associations between friend characteristics and HIV could be biased, mostly likely downward.

We therefore sought to separate out those young women who had acquired HIV through sexual transmission and those who had been infected via MTCT (mother to child transmission).

#### Methods

We did not have means of knowing with certainty which young women might have been infected via MTCT. However, there were 36/76 young women who were HIV positive and who reported never having had sex. While it is possible that ever sex was under‐reported, the study did not have other data to determine non‐sexual HIV infection.

We first compared the socio‐demographic characteristics of young women who were HIV positive by whether or not they reported ever having had sex and used χ^2^ tests and *t* tests to examine whether there was statistical evidence for differences. Young women who were vertically infected were expected to be more likely to be double or single orphans (via mother's infection) and younger. If they had been HIV positive for most of their lives they were also expected to be more likely to have had interrupted schooling and therefore to have been in a lower school grade for their age.

We then compared the associations observed between participant and friend characteristics and HIV including all young women (n=2326), and excluding those who were HIV positive but reported never having had sex (n=2290). We also compared the findings of these models to those examining the association between friendship characteristics and participant ever sex and participant HSV‐2 status. Risk of being HSV‐2 positive was hypothesised to share similar causal pathways and to have similar patterns of associations with sexually acquired HIV, but not non‐sexually acquired HIV.

### Results

#### Characteristics of HIV‐positive participants by reported ever sex

There were 36/76 (47.4%) of young women who were HIV positive but reported never having had sex, (Table 4). Comparing these young women with those who were HIV positive but who did report ever having had sex, those reporting never sex were less likely to be HSV‐2 positive (8.3% compared to 37.5%, *p* = 0.003). Those reporting never sex were also younger (mean age =15.0 rather than 17.4, *p* < 0.001) and in lower school grades (58.3% in Grade 8 compared to 5.0%, *p* < 0.001). There was little evidence for a difference between groups by father's education or household socio‐economic position, but young women who were HIV positive and reporting never sex tended to have higher educated mothers (8.3% had no school compared to 20.0% of those who reported ever sex). While there was a higher proportion of young women who were HIV positive and reported ever having had sex who had both parents alive (55.0% vs. 44.4% who reported never having had sex) and more of those reporting never sex were double orphans (25.0% vs. 15.0%), there was little evidence that the difference in orphanhood status was more than might be expected by chance (*p* = 0.706).

**Table 4:** Characteristics of HIV‐positive participants by whether they report ever having had sex, n=76

Participant characteristics	HIV‐positive reporting never sex, n=36		HIV‐positive, reporting ever sex, n=40		*p* value
	n	%	n	%	
HSV2 status					
Negative	33	91.7	25	62.5	0.003
Positive	3	8.3	15	37.5	
Age in years	15.0	(mean)	17.4	(mean)	<0.001
School grade					
8	21	58.3	2	5.0	<0.001
9	6	16.7	8	20.0	
10	5	13.9	11	27.5	
11	4	11.1	19	47.5	
Orphanhood					
Both parents alive	16	44.4	22	55.0	0.706
Mother only alive	8	22.2	9	22.5	
Father only alive	3	8.3	3	7.5	
Neither parent alive	9	25.0	6	15.0	
Household SEP					
First quintile (lowest)	9	25.0	10	25.0	0.915
Second	6	16.7	7	17.5	
Third	7	19.4	6	15.0	
Fourth	7	19.4	11	27.5	
Fifth	7	19.4	6	15.0	
Mother's education					
No school	3	8.3	8	20.0	0.027
Attended primary but not completed	9	25.0	13	32.5	
Completed primary, some high school	7	19.4	10	25.0	
Completed high school	15	41.7	4	10.0	
Do not know	2	5.6	5	12.5	
Father's education					
No school	6	16.7	8	20.0	0.832
Attended primary but not completed	6	16.7	6	15.0	
Completed primary, some high school	3	8.3	5	21.5	
Completed high school	14	38.9	11	27.5	
Do not know	7	19.4	10	25.0	

#### Associations between HIV and friendship characteristics amongst all HIV‐positive young women

When including all young women in the analyses (n=2326), the strength of associations between HIV and young women's socio‐demographic characteristics and those of their friends with HIV reduced compared to when positive young women reporting never sex were excluded (OR=1.31, 95% CI 1.15 to 1.49 compared to OR=1.89, 95% CI 1.57 to 2.28 excluding HIV‐positive young women reporting never sex, Table 5). There was a much strengthened association with school grade, whereby being HIV positive were less likely to be in grades 9, 10 and 11, even adjusted for age and other characteristics. Orphanhood status was also associated with HIV, such that young women for whom both parents had died had 4.67 times the odds (95% CI 2.48 to 8.78) of being HIV positive compared to young women for whom both parents were alive. When excluding the young women who reported never having had sex but who were HIV positive, the strength of this association reduced (OR=2.78, 95% CI 1.08 to 7.20)

**Table 5:** Associations between participant and friendship characteristics when excluding HIV‐positive young women and reporting never sex (n=2290) and when including all young women (n=2326), adjusted for participant age

	Excluding those HIV positive reporting never sex, n=2290				Including all young women, n=2326			
	OR	95% CI		*p* value	OR	95% CI		*p* value
Each additional year in age	1.89	1.57	2.28	<0.001	1.31	1.15	1.49	<0.001
School grade								
8	1.00			0.618	1.00			0.005
9	2.11	0.44	10.18		0.39	0.19	0.78	
10	1.32	0.27	6.45		0.27	0.13	0.57	
11	1.84	0.38	9.07		0.36	0.17	0.80	
Orphanhood								
Parents alive	1.00			0.277	1.00			<0.001
Mother only alive	1.25	0.56	2.76		1.47	0.82	2.64	
Father only alive	1.43	0.41	4.97		1.76	0.73	4.26	
Neither parent alive	2.78	1.08	7.20		4.67	2.48	8.78	
Household SEP								
First quintile	1.00			0.631	1.00			0.739
Second	0.74	0.27	2.00		0.71	0.34	1.45	
Third	0.67	0.24	1.89		0.72	0.35	1.47	
Fourth	1.36	0.56	3.30		1.01	0.52	1.96	
Fifth	0.79	0.28	2.23		0.75	0.36	1.54	
Mother's education								
No school	1.00			0.458	1.00			0.609
Attended primary but not completed	1.38	0.56	3.42		1.64	0.78	3.43	
Completed primary, some high school	0.95	0.37	2.46		1.05	0.48	2.27	
Completed high school	0.52	0.15	1.78		1.39	0.65	2.99	
Do not know	1.38	0.44	4.38		1.32	0.50	3.47	
Father's education								
No school	1.00			0.728	1.00			0.292
Attended primary but not completed	0.75	0.25	2.21		0.86	0.39	1.89	
Completed primary, some high school	0.86	0.27	2.70		0.61	0.25	1.47	
Completed high school	1.38	0.54	3.55		1.34	0.68	2.64	
Do not know	1.29	0.50	3.37		1.21	0.59	2.50	
Each additional friend perceived to have had sex	1.36	1.09	1.68	0.004	1.03	0.96	1.11	0.406
Each additional friend ≥1 year older	1.44	1.14	1.83	0.005	1.11	0.92	1.34	0.293
No male friends				0.135	1.00			0.028
≥1 male friend	1.86	0.86	4.01		1.93	1.11	3.37	
All friends in school	1.00			0.112	1.00			0.003
≥1 friend out of school	1.74	0.89	3.41		2.15	1.31	3.54	
Each additional friend who is a relative	1.09	0.97	1.23	0.157	1.06	0.97	1.16	0.194

Including all young women, there was still strong evidence for an association between friendship characteristics and HIV, but these associations differed from when HIV‐positive young women reporting never sex were excluded. Evidence for association between HIV and each friend at least one year older diminished to OR=1.11, 95% CI 0.92 to 1.34) and weakened considerably for each friend perceived to have had sex reduced to OR=1.03 (95% CI 0.96 to 1.11) from 1.36 (95% CI 1.09 to 1.68). Instead, there was strengthened evidence that young women with at least one friend out of school (OR=2.15, 95% CI 1.31 to 3.54) and at least one male friend (OR=1.93, 95% CI 1.11 to 3.37) were more likely to be HIV positive.

### Discussion

Compared to HIV‐positive participants reporting ever having had sex, those reporting never sex were less likely to be HSV‐2 positive, younger and in a lower grade at school, though not more likely to be orphans.

When including all HIV‐positive women, associations between participant characteristics and HIV status changed such that associations with age weakened and associations with being in a lower grade for age and with having neither parent alive emerged and strengthened respectively. Associations were more to similar to those seen with HSV‐2 (Tables 2 and 3), a sexually transmitted infection, when excluding HIV‐positive young women who reported never having had sex.

Young women who were perinatally infected would have been HIV positive for longer and would be more likely to have been ill enough to have missed or interrupted their schooling and so might be more likely to be in a lower grade for their age, although this has also been found to be a result of orphanhood 68. It is more likely that they would be younger and that their own parents would have died than young women who acquire HIV as a sexually transmitted infection. That these factors were not associated with HSV‐2 infection lends further support to interpreting these young women as perinatally infected.

It is likely that long‐term HIV infection would affect a young woman's friendships. Cross‐sectional associations could reflect this, leading to different results when including the possibly perinatally infected young women. It is plausible that young women who had been HIV infected for a long time might be excluded from the predominantly female, in‐school friendship network and thus more likely to have male and out‐of‐school friends instead. However, it is possible that the improved evidence for these associations related partly to differences in power between models including and excluding HIV‐positive girls reporting never having had sex. It would not be appropriate to draw conclusions about this pathway from the data we have, but this is an area for further exploration, given the importance of social support for HIV‐positive adolescents 69, 70, 71.

### Conclusions

While recognising that there is likely to be bias in the reporting of ever sex, and that we are unable to distinguish perinatal from sexually transmitted HIV infections with certainty, our findings are consistent with the idea that many of the young women who were HIV positive yet reported never having had sex were indeed not sexually infected. When investigating the role of friendships on the causal pathways related to sexual transmission we therefore decided that it was most appropriate to exclude these young women from primary HIV analyses.
